# In vivo evaluation of a vibration analysis technique for the per-operative monitoring of the fixation of hip prostheses

**DOI:** 10.1186/1749-799X-4-10

**Published:** 2009-04-09

**Authors:** Leonard C Pastrav, Siegfried VN Jaecques, Ilse Jonkers, Georges Van der Perre, Michiel Mulier

**Affiliations:** 1 Division of Biomechanics and Engineering Design (BMGO), Katholieke Universiteit Leuven, Celestijnenlaan 300C, bus 2419, 3001 Heverlee, Belgium; 2 Group T Leuven Engineering College (Association K.U. Leuven), Vesaliusstraat 13, 3000 Leuven, Belgium; 3 BIOMAT Research Cluster, Katholieke Universiteit Leuven, Kapucijnenvoer 7, 3000 Leuven, Belgium; 4 Dept Biomedical Kinesiology, Katholieke Universiteit Leuven, Tervuursevest 101, 3000 Leuven, Belgium; 5 Department of Orthopaedics, Katholieke Universiteit Leuven, Weligerveld 1, 3212 Lubbeek, Belgium

## Abstract

**Background:**

The per-operative assessment of primary stem stability may help to improve the performance of total hip replacement. Vibration analysis methods have been successfully used to assess dental implant stability, to monitor fracture healing and to measure bone mechanical properties. The objective of the present study was to evaluate in vivo a vibration analysis-based endpoint criterion for the insertion of the stem by successive surgeon-controlled hammer blows.

**Methods:**

A protocol using a vibration analysis technique for the characterisation of the primary bone-prosthesis stability was tested in 83 patients receiving a custom-made, intra-operatively manufactured stem prosthesis. Two groups were studied: one (n = 30) with non cemented and one (n = 53) with partially cemented stem fixation. Frequency response functions of the stem-femur system corresponding to successive insertion stages were compared.

**Results:**

The correlation coefficient between the last two frequency response function curves was above 0.99 in 86.7% of the non cemented cases. Lower values of the final correlation coefficient and deviations in the frequency response pattern were associated with instability or impending bone fracture. In the cases with a partially cemented stem an important difference in frequency response function between the final stage of non cemented trial insertion and the final cemented stage was found in 84.9% of the cases. Furthermore, the frequency response function varied with the degree of cement curing.

**Conclusion:**

The frequency response function change provides reliable information regarding the stability evolution of the stem-femur system during the insertion. The protocol described in this paper can be used to accurately detect the insertion end point and to reduce the risk for intra-operative fracture.

## Background

Total hip replacement (THR) is the second most performed surgical procedure with an estimated number of more than one million operations each year worldwide. This implies that, despite survival rates of 97% at 3 years [1] and even up to 10 years follow-up [2] for some prosthesis types, a large number of revision operations are needed every year, most of them because of aseptic loosening. Revision operations are more difficult to perform, carry more risk for complications and have a poorer prognosis than primary THR [3].

Survival rate is directly related to the long term fixation stability of the prosthesis stem [4]. Beside the design, material composition and surface characteristics of the implant, the initial per-operative fixation of the stem in the femoral bone has a critical influence on its long term fixation stability. This is especially the case for non cemented, press-fit fixated stems. The insertion procedure results in well-defined contact areas and interface pre-stresses between the stem and the femoral bone. Under actual loading, the hip stem displacement and the femoral stress distribution will strongly depend upon these initial contact conditions. Primary hip stem stability is not only important regarding prosthesis migration, but also regarding micro movements that must be limited in order to allow interfacial bone formation and in-growth [5]. Femoral stress distribution has a crucial influence on bone remodelling and therefore on the final strength of the bone-implant structure. Therefore the per-operative characterization of the primary stem-femur contact and the assessment of primary stem stability in the first place may help to improve the survival rate of THR.

Nowadays objective intra-operative assessment of primary stem stability is a challenge, as surgeons have to rely mainly on their clinical experience, which consists mainly of a sense of mechanical stability when exerting axial force and/or torque on the prosthesis. Moreover, excessive press-fitting of a THR femoral component can cause intra-operative fractures with an incidence of up to 30% in revision cases [6].

Vibration analysis has been successfully used to determine bone mechanical properties [7-9]. Clinical applications of this method were monitoring of fracture healing and in vivo assessment of bone mechanical properties [10-14]. Vibration analysis was also successfully used to quantify the fixation of oral implants [15]. A limited number of studies prove the feasibility of detecting several forms of femoral implant loosening, in vitro and in vivo using techniques based on harmonic distortion [16-19].

In vitro, the analysis of frequency response function (FRF) was used to discriminate between well fixed and quasi-well fixed femoral stems [20].

This paper presents a series of cases where a per-operative vibration analysis technique was used for the mechanical characterization of the primary bone-prosthesis stability.

In a previous study we demonstrated the feasibility and validity of a vibration analysis technique for the assessment of the femur-stem fixation in vitro [21-24]. The stem insertion process was performed on a dry cadaver femur and synthetic composite femurs and the FRF change was analysed. In a recent study a finite element model was created to gain insight into the dependence of the FRF on system parameter variations [25].

The imperfections in the connection between a THR prosthetic stem and a femur can most sensitively be detected by observing shifts in the resonance frequency of the higher vibration modes of the femur-prosthesis system. This observation is in accordance with the work of Qi et al. who stated that the most sensitive frequency band for observing defects in the femur-prosthesis connection is above 2500 Hz [26].

In the present study the vibration analysis technique was applied for the per-operative assessment of fixation stability in 83 THR patients who obtained an intra-operatively manufactured prosthesis (IMP) provided by Advanced Custom Made Implants, Leuven, Belgium (see appendix 1). The IMP approach aims at optimal stem stability through a maximum fit and fill of the femoral cavity [27].

The objective of the present study was to apply and evaluate an endpoint criterion for the insertion of the stem by successive surgeon-controlled hammer blows. The endpoint-of-insertion criterion was based upon the Pearson's correlation coefficient R between the FRFs of two successive insertion stages.

## Methods

From the previous *in vitro *studies a protocol was derived to be applied in per-operative conditions.

The prosthesis neck was attached to a shaker (Bruel & Kjaer, Naerum, Denmark, model 4810) using a stinger provided with a clamping system. The excitation was realized through white noise in the range 0–12.5 kHz. The input force and the response acceleration were measured in the same point with an impedance head (PCB Piezotronics, Depew, New York, USA, model nr 288D01) mounted between the shaker and the stinger. The stinger is a slender rod threaded at both ends, one of them being connected to the impedance head and the other one to a kind of claw forming a clamping system that is firmly attached to the prosthesis neck.

**Figure 1:**
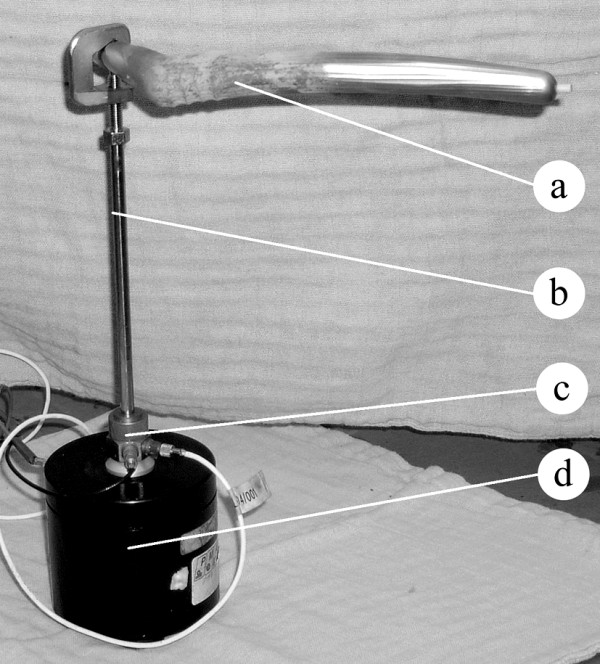
**Experimental setup**. a. Hip stem. b. Stinger and clamping system. c. Impedance head. d. Shaker.

The excitation system used low amplitude vibrations and introduced approximately 0.5 W of power into the femur-prosthesis system. This is considered as safe and no adverse effects have been reported by other authors using a similar excitation system [11-13]. The experimental setup is shown in Figure 1.

**Figure 2:**
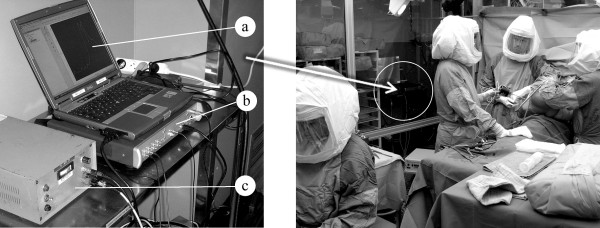
Measuring hardware (left) and surgical theatre (right*). a. Portable computer. b. Vibration analyser Pimento^®^. c. Power amplifier. *The circle indicates the place of the measuring hardware (presented in the left picture) behind the transparent wall. A member of the surgical team holds the shaker during the insertion procedure.

The FRF was measured and recorded by a Pimento vibration analyser (LMS International, Haasrode, Belgium) connected to a portable computer provided with the appropriate software (Pimento 5.2, LMS International, Haasrode, Belgium). The vibration analyser generates the excitation signal which is amplified and sent to the shaker. The vibration analyser, the portable computer and the amplifier were installed in the surgical theatre but outside the so-called laminar flow area (Figure 2).

Patients, eligible for THR, received full information relative to the surgical intervention and the study objectives, including the scheme for follow-up visits. The study protocol was approved by the institutional review board. Patients were included after giving written informed consent. Thirty patients received non cemented IMP stems and fifty three patients received distally cemented IMP stems. The decision between the two procedures was made by the surgeon on clinical criteria. All stems were proximally coated with hydroxyapatite.

Before starting the measurements on patients the full protocol was tested in a cadaver study.

### Non cemented prostheses

The surgeon inserted the implant in the femoral canal through successive controlled hammer blows. After each blow, the FRF of the implant-bone structure was measured directly on the prosthesis neck in the range 0–10 kHz.

**Figure 3:**
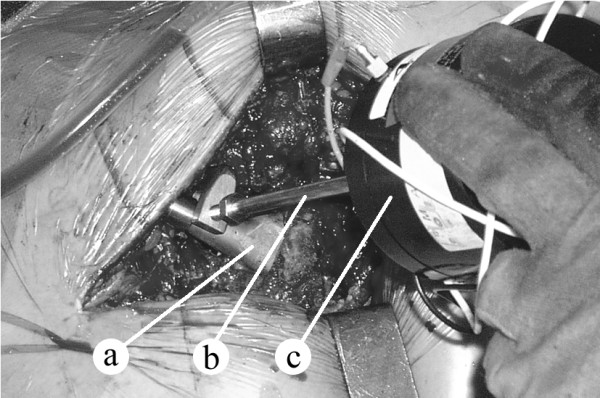
**Hip stem insertion**. a. Hip stem (almost fully inserted). b. Stinger and clamping system. c. Shaker (held by a member of the surgical team).

During the insertion the assembly composed by shaker, impedance head and stinger with clamping system was all the time attached to the prosthesis neck (i.e. the clamping was done only once per insertion, and the tightness of clamping was thus the same for all FRFs i = 0...n). In the measured structure the only variable was the connection between the implant and the bone. The shaker was held by a member of the surgical team as presented in Figure 2 (right) and Figure 3.

The FRF changes were used as indicators of the evolution of the stiffness of the implant-bone structure and, as a consequence, the evolution of the implant stability. When the FRF graph did not change noticeably anymore the hammering was stopped. Extra blows would not improve the stability of the prosthesis but would increase the fracture risk.

The similarity of two successive FRF graphs was evaluated using the Pearson's correlation coefficient. A correlation between the FRFs of successive stages of R = (0.99 +/- 0.01) over the range 0–10000 Hz is proposed as an endpoint criterion.

### Partially cemented prostheses

The per-operative protocol presented above was adapted to assess the stability of hybrid IMPs that were partially cemented distally using Palacos^® ^(Zimmer Inc. Warsaw, Indiana, USA) bone cement [27].

In a first stage, the surgeon inserted the stem completely in the femur without cement, for a trial reduction of the artificial joint. In a later stage, the stem was removed, cement was introduced in the distal part of the femoral canal, the stem was re-introduced and after the cement has fully cured, the implant was supposed to be completely fixed. The FRF was measured in both stages using the same method as in the non cemented stems case. (Figure 3). In some randomly chosen cases, the FRF was measured also at various stages of cement curing i.e. 6, 10, 12, and 14 minutes after cement preparation.

## Results

### Non cemented hip stems

**Figure 4:**
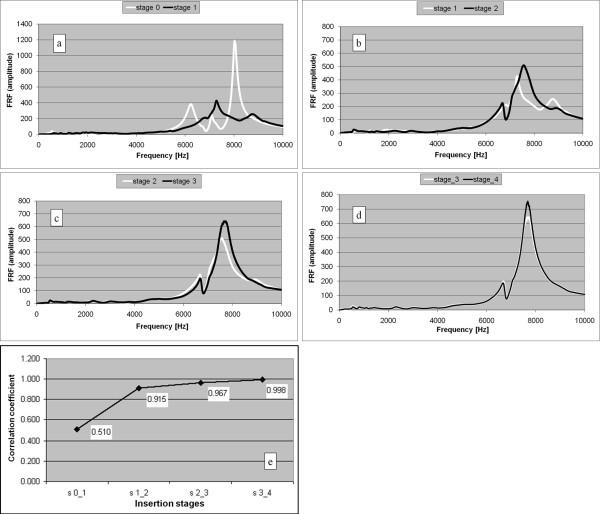
**Non cemented stem**. a-d. FRF graphs corresponding to successive insertion stages. e. Pearson's correlation coefficients calculated for the FRF pairs presented in Figures 4 a-d. For example, the point with the abscissa "s 0_1" and the ordinate "0.510" represents the correlation coefficient calculated between the FRFs corresponding to the insertion stages 0 and respectively 1. The graphs corresponding to these FRFs are presented in Figure 4a.

Thirty cases of non cemented stems were studied *in vivo *and a typical evolution of the FRF graph is shown in Figures 4a–d. The Pearson's correlation coefficient (R), calculated for consecutive pairs of FRFs, is presented in Figure 4e. Stage 0 corresponds to the FRF calculated after the stem was introduced in the femur by hand; stage 1 corresponds to the FRF calculated after the first hammer blow series, stage 2 after the second hammer blow series and so on. The surgeon needed five stages (0...4) to completely insert the stem in this case.

Normally, the FRF graphs shifted to the right indicating a stiffness increase between successive insertion stages [28]. To compare the similarity of two successive FRF graphs the Pearson's correlation coefficient was used. Due to the fact that there is no linear dependence of one graph with respect to the other, the two graphs are identical if the correlation coefficient is 1.

In twenty six out of thirty cases (86.7%), the correlation coefficient between the last two FRFs was above 0.99 when the surgeon stopped the insertion. In the other four cases, when the surgeon decided to stop the insertion because of suspected bone fragility, the final correlation coefficient reached lower values still exceeding 0.95.

### Non cemented hip stems – non-typical cases

#### Case 1

**Figure 5:**
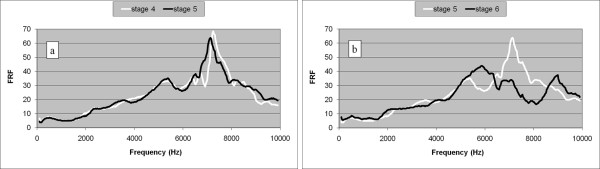
**Non cemented stem in human cadaver (non-typical case 1)**. a. FRF graphs corresponding to the insertion stages 4 and 5. b. FRF graphs corresponding to the insertion stages 5 and 6.

While testing the per-operative protocol on a human cadaver, the stem was deliberately inserted until the femur was fractured. The last three FRF graphs are presented in Figures 5a and 5b.

The FRF graph slightly shifted to the left at the fifth insertion stage indicating a decrease of the stability before the sixth stage when the bone was fractured. The final FRF graph is totally different with respect to the previous graph indicating an important change in the stem-femur structure.

#### Case 2

**Figure 6:**
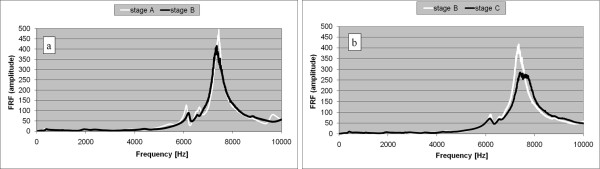
**Per-operative fracture (non-typical case 2)**. a. FRF graphs corresponding to the insertion stages A and B (anomalous left shift). b. FRF graphs corresponding to the insertion stages B and C (small fracture).

During a per-operative experiment, when the stem was quasi fully inserted, the highest peak of the FRF graph slightly shifted to the left (stage B in Figure 6a).

After a supplementary hammer blow series, the corresponding FRF graph presented an abnormal shape (stage C in Figure 6b). Inspecting the bone, a small fracture was observed and the hammering was stopped.

#### Case 3

**Figure 7:**
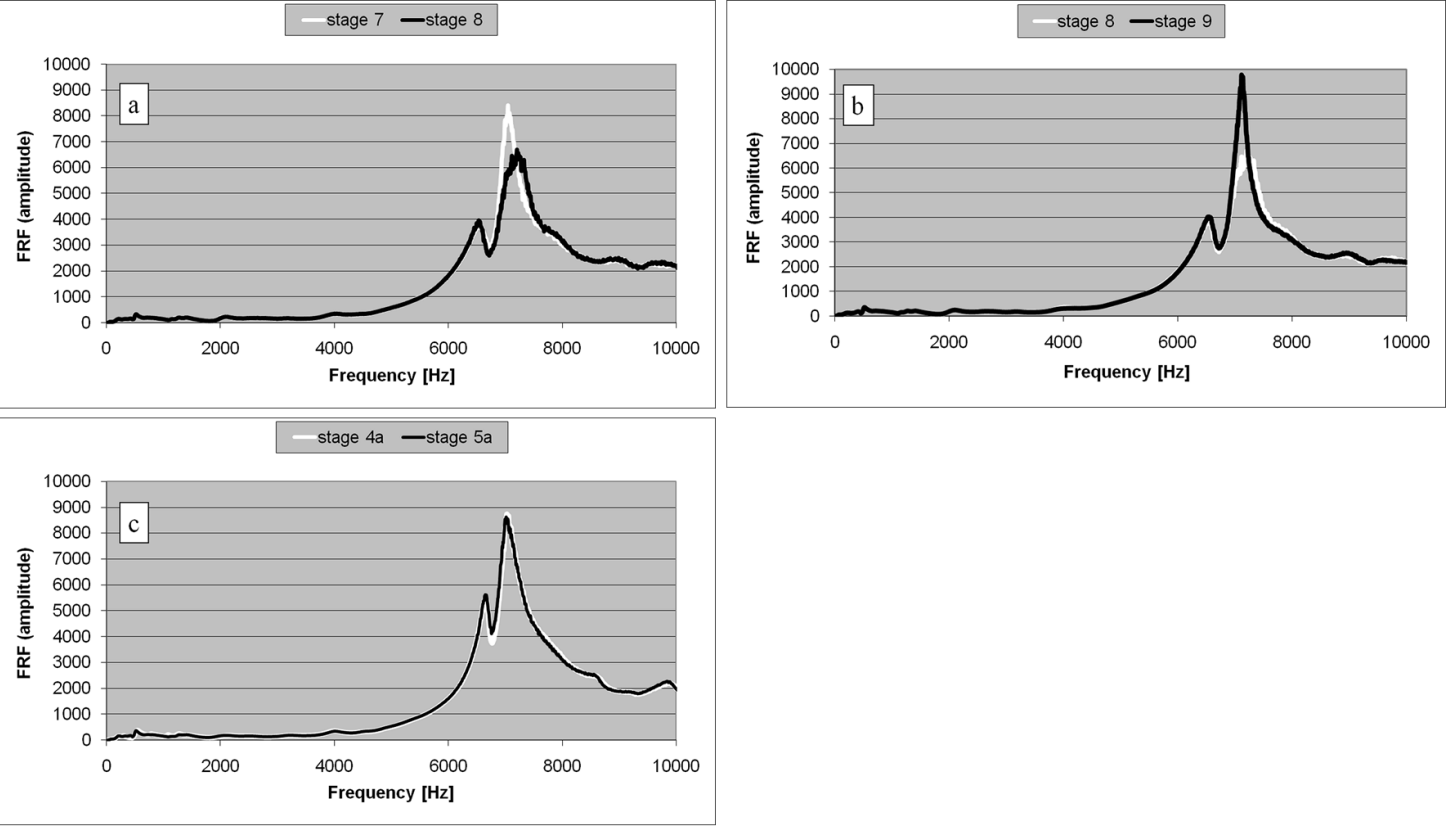
**Correction of the femoral canal (non-typical case 3)**. a. FRF graphs corresponding to the insertion stages 7 and 8 (typically observed right shift). b. FRF graphs corresponding to the insertion stages 8 and 9 (anomalous left shift). c. FRF graphs corresponding to the two final stages (labelled 4a and 5a) of the reinsertion process, after the correction of the femoral canal.

An oscillating behaviour of the FRF graph was observed during another per-operative hip arthroplasty procedure (stages 7, 8, and 9 in Figures 7a and 7b).

Since the stem was visibly not fully inserted, the hammering normally had to continue, but the behaviour of the FRF, similar to the FRF evolution presented in case 2, was indicating that the stem was blocked and, as a consequence, there was a risk for fracture. The problem was solved by pulling out the stem, adjusting the femoral canal and reinserting the prosthesis. The FRF had a normal evolution during the reinsertion and the graphs corresponding to the final two stages, labelled as stage 4a and stage 5a, are shown in Figure 7c. The corresponding Pearson's correlation coefficient attained 0.998.

### Partially cemented hip stems

**Figure 8:**
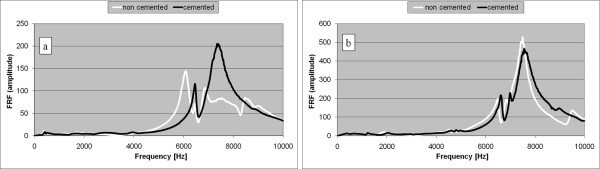
**Two typical cases of partially cemented stems completely inserted in the femur**. a. FRF graphs for two stages: without cement (white) and cemented (black). An important change can be observed after cementation. b. FRF graphs for two stages: without cement (white) and cemented (black). FRF graph slightly shifted to the right after cementation.

Fifty three cases of partially cemented prostheses were studied in vivo. In forty five cases (84.9%) an important difference was observed between the FRF graph corresponding to the non cemented stage and the FRF graph corresponding to the cemented stage, after complete cement hardening, in frequency and amplitude. A typical example is shown in Figure 8a.

In the other eight cases, although some alteration could be noticed, the FRF graph did not substantially change after cement curing (Figure 8b).

**Figure 9:**
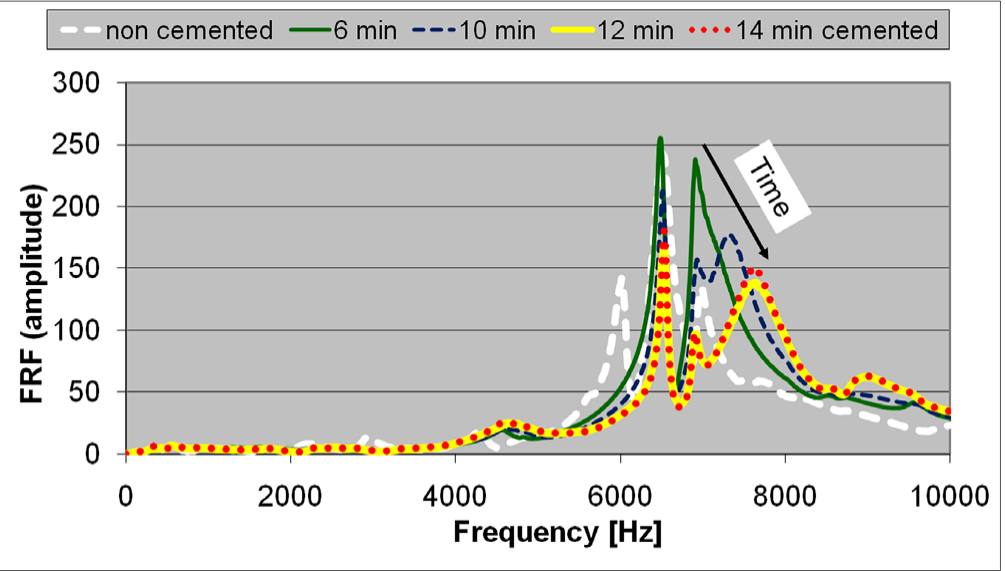
**FRF graphs of a typical stem-femur system during cement curing**. The evolution of the FRF at various stages of cement curing i.e. 6, 10, 12, and 14 minutes after cement preparation. The arrow indicates that the peak corresponding to the vibrational mode mostly influenced by the cement curing shifts to the right when the curing time increases.

The typical evolution of the FRF graph during the cement curing, at 6, 10, 12, and 14 minutes after cement mixing, is shown in Figure 9.

When the cement polymerisation sets in, the resonance frequencies of the vibration modes associated with the cement increase. For the presented case, the resonance frequency of the vibration mode mostly influenced by the cement curing increased from 7180 to 7680 Hz. When the polymerisation is complete, no further changes are observed in the FRF. The graphs corresponding to 12 and 14 minutes are nearly identical.

## Discussion

During the insertion of an IMP in a femur, the changes of boundary conditions and implant stability between different stages are reflected by the FRF evolution as observed per-operatively. The higher resonance frequencies are more sensitive to the stability change than the lower frequencies. This observation is in accordance with previous finite element studies [26], and can be explained schematically as follows. The lower frequency resonances correspond to vibration motions in which the deformation of the femur (vibration mode shape) is simple, such as single bending of the femur shaft. The higher frequency resonances correspond to more intricate deformation modes of the femur in combination with deformation modes of the prosthesis. In the case of simple bending modes of the femur the prosthesis stem acts just like an added mass and its influence depends more on its position than on the fixation conditions. In the case of more intricate mode shapes the interaction between the stem and the femur becomes more complicated and the corresponding resonance frequencies become more sensitive to the interface conditions. This explanation is completely corroborated and further elucidated by recent advanced finite element analyses by our group [25,29].

During the insertion of the uncemented stem the FRF change is influenced by the stiffness of the implant-femur system and the relative position of the two components. The FRF graph shift to the right between successive hammer blows is a normal evolution; the fixation stiffness increase being reflected by increasing resonance frequencies. The graph change in shape and position is more important at the beginning of the insertion when the stem displacement is important as well. At the end of the insertion, when the resistance against the stem displacement increases, the shapes of the successive graphs are very similar and the shift is less important. When the FRF graph does not change noticeably between two hammer blows, the logical conclusion is that the system mechanical parameters do not change, thus the stem cannot move and the hammering should stop to avoid an intra-operative fracture.

For non cemented stems, the Pearson's correlation coefficient between successive FRFs can be used as a criterion for the detection of the insertion endpoint.

Moreover, the FRF analysis can be used to detect dangerous situations during surgery such as stem blockage and fracture risk. An FRF graph shift to the left indicates a decreasing fixation, probably due to plastic deformation of the bone, and should be a serious warning for the surgeon. In two cases hammering after a graph shift to the left resulted in bone fractures (Figures 5b and 6b).

A possible fracture may have been avoided in the case of an abnormal bone structure and a deformed endomedullary canal as the FRF analysis showed an abnormality and the surgeon was alerted to the situation in time during insertion of the stem (Figures 7a–c).

The supplementary information obtained by vibration analysis helps the surgical team to take the optimal decisions.

The curing of bone cement in partially cemented hip stem systems can also be monitored by vibration analysis.

In 15% cases the FRF graph did not substantially change after cement curing. The interpretation could be that the implant stability did not considerably change after cementation. Probably the stems were already reasonably well fixed in the non cemented stage. However, the shift to the right of the FRF graph indicates an increased stability after cementation. Comparing the Figures 8a and 8b, it can be observed that the FRF corresponding to the complete curing stages (black graphs) are very similar. Moreover, these graphs are very similar to the graphs corresponding to the final insertion stage of the cementless stems (Figures 4d and 7c)

The per-operative experimental study should be completed and validated by an appropriate post-operative follow-up of the patients. In an ongoing clinical study, part of project OT/03/31, migration of the stems is followed up by Roentgen Stereophotogrammetric Analysis (RSA) and bone remodelling is followed up by Dual energy X-Ray Absorptiometry (DXA). Conventional follow-up by clinical examination, radiographs and standardised questionnaires is also part of the protocol [30,31].

## Conclusion and future work

The presented per operative technique was designed to monitor the stability and to detect the insertion endpoint of non cemented and partially cemented hip stems, but it can be adapted for other orthopaedic implants as well.

It does not provide direct quantitative information on the displacements in real life loading conditions, but it is a powerful technique for the quality and safety control of the surgical procedure. It is a sensitive, minimally invasive method to check whether the insertion process runs normally and results in the best possible fixation for the patient and the prosthesis at hand, and to prevent bone fracture.

The stability under real life loads of an optimally fitted non cemented prosthesis such as the IMP and other prosthesis systems has been shown to be adequate by previous research and clinical experience.

Nevertheless vibration analysis is currently developed into a technique for the full mechanical characterization of the contact between the prosthesis and the bone in terms of contact areas, interface stresses and ultimately stability under real loading. In a finite elements study [25] the relation between the vibration behaviour and the spatial distribution of contact areas was analyzed. In a transient dynamic analysis [29] the successive steps in the insertion process were simulated in terms of contact areas and interface stresses, and the vibration response in each step was calculated by finite elements analysis.

Building on the understanding and clinical experience built through per operative monitoring, vibration analysis will be developed further into a technique for the non invasive post operative assessment of prosthesis fixation, in view of detection of loosening.

## Competing interests

The authors declare that they have no competing interests.

## Authors' contributions

All authors have made substantial contributions to the conception and design of the study, analysis and interpretation of data, drafting the article, and revising it critically. Specifically, LP developed the details of the vibration analysis protocol, operated the data acquisition equipment during the peroperative measurements, processed and analysed the FRF data (supervised by GVdP and SJ) and drafted the figures and the initial version of the manuscript. GVdP and SJ conceived the principles of the vibration analysis protocol. GVdP, IJ and SVNJ drafted the grant application from which this study was partially funded, including the study design, and supervised the implementation. MM was the surgeon in charge during the THR procedures and operated the sterile part of the vibration analysis equipment within the laminar flow area. MM provided clinical background knowledge for the introduction and discussion sections. Multiple critical iterations on the initial manuscript were a joint effort of all authors. All authors read and approved the final manuscript.

## Appendix

### The intra-operatively manufactured prosthesis

In the IMP-procedure, the stem of the prosthesis is custom made for each individual patient, during the operation [27]. After reaming of the femoral cavity, a 3D imprint of the cavity is made in the form of a silicone mould. This mould is scanned and its geometry is optimized using a CAD-procedure. Based upon this adapted geometry, a CNC milling machine then transforms a partially preformed prosthesis stem into the final personalized shape. Using this technique a maximum fit and fill of the femoral cavity might be obtained allowing an optimal stability of the femoral implant.

Between the first implantation in 1987 and the time of writing (2009) the technique underwent some changes, the most important of which was the application of a hydroxyapatite layer by plasma spray coating.

### Pearson's correlation coefficient

Pearson's product moment correlation coefficient, *R*, is a dimensionless index that ranges from -1.0 to 1.0 inclusive and reflects the extent of a linear relationship between two data sets (i.e. two variables).

The equation for the correlation coefficient is:



Where *x *and *y *are the two variables and ,  are the corresponding arithmetic means [32].
